# Evolutionary History and Functional Diversification of the *JmjC* Domain-Containing Histone Demethylase Gene Family in Plants

**DOI:** 10.3390/plants11081041

**Published:** 2022-04-12

**Authors:** Shifeng Ma, Zhiqiang Zhang, Yingqiang Long, Wenqi Huo, Yuzhi Zhang, Xiaoqing Yang, Jie Zhang, Xinyang Li, Qiying Du, Wei Liu, Daigang Yang, Xiongfeng Ma

**Affiliations:** 1 Zhengzhou Research Base, State Key Laboratory of Cotton Biology, School of Agricultural Sciences, Zhengzhou University, Zhengzhou 450001, China; msf2411532161@gs.zzu.edu.cn (S.M.); 202012582017548@gs.zzu.edu.cn (Y.L.); 202022582017522@gs.zzu.edu.cn (W.H.); zhangjie2935@163.com (J.Z.); d202122582017772@gs.zzu.edu.cn (Q.D.); 2 State Key Laboratory of Cotton Biology, Institute of Cotton Research, Chinese Academy of Agricultural Sciences, Anyang 455000, China; 82101199208@caas.cn (Z.Z.); 82101202203@caas.cn (X.Y.); 82101211074@caas.cn (X.L.); 3 Collaborative Innovation Center of Henan Grain Crops, Agronomy College, Henan Agricultural University, Zhengzhou 450002, China; zyz2020@stu.henau.edu.cn

**Keywords:** histone demethylation, *JmjC* gene, phylogeny, growth and development, stress response

## Abstract

Histone demethylases containing JumonjiC (*JmjC*) domains regulate gene transcription and chromatin structure by changing the methylation status of lysine residues and play an important role in plant growth and development. In this study, a total of 332 *JmjC* family genes were identified from 21 different plant species. The evolutionary analysis results showed that the *JmjC* gene was detected in each species, that is, the gene has already appeared in algae. The phylogenetic analysis showed that the KDM3/JHDM2 subfamily genes may have appeared when plants transitioned from water to land, but were lost in lycophytes (*Selaginella moellendorffii*). During the evolutionary process, some subfamily genes may have been lost in individual species. According to the analysis of the conserved domains, all of the plant *JmjC* genes contained a typical JmjC domain, which was highly conserved during plant evolution. The analysis of *cis*-acting elements showed that the promoter region of the *JmjC* gene was rich in phytohormones and biotic and abiotic stress-related elements. The transcriptome data analysis and protein interaction analyses showed that *JmjC* genes play an important role in plant growth and development. The results clarified the evolutionary history of *JmjC* family genes in plants and lay the foundation for the analysis of the biological functions of *JmjC* family genes.

## 1. Introduction

Epigenetics is a discipline that studies on the changes in gene expression, which can be heritable changes in without changes in the DNA sequence [1,2]. Gene expression is regulated by epigenetics, including DNA methylation, histone modification, and chromatin remodeling. Non-coding RNA plays a key role in the regulation of chromatin structure and dynamic gene expression [3,4,5]. Histone lysine methylation plays an important role in the epigenetic regulation of gene expression in multicellular eukaryotes (including plants) [6]. Histone methylation mainly occurs at the N-terminal lysine (K) and arginine (R) sites of histones H3 and H4, including K4, K9, K27, K36, K79 of H3; K20 of H4; and R2, R17, R26 of H3, R3 of H4. The methyl group on S-adenosylmethionine is transferred to the guanidine group at the end of target protein lysine residue by histone methyl transferase (HMT). Lysine can undergo three types of methylation, namely, monomethylation (Kme1), dimethylation (Kme2), or trimethylation (Kme3) modification, while arginine can only undergo monomethylation (Rme1) modification, symmetric dimethylation (Rme2s) modification, or asymmetric dimethylation (Rme2a) modification [7]. The methylation of histone lysine has a variety of effects, not only affecting the electrostatic charge of the modified residues, but also improving the hydrophobicity and changing the intramolecular or intermolecular interactions [8]. In addition, histone lysine methylation modification at different sites has different effects on transcriptional regulation. The methylation of histones H3K4, H3K36, and H3K79 is mainly concentrated in the promoter region of active transcription, so it is related to the transcription activation of genes; the methylation of H3K9 can be used as a heterochromatin marker and inhibit gene transcription. The methylation of H3K27 is associated with gene silencing [9].

According to the different modes of action, the two histone lysine demethylases that play an important role in the demethylation of lysine residues in organisms are divided into two categories: the first is histone lysine Acid-specific demethylase 1 (LSD1), which can reverse the monomethylation or dimethylation of lysine residues through flavin adenine dinucleoside FAD (Flavin adenine dinucleotide), but does not act on trimethylated lysine [10]. The second histone lysine demethylase, JHDM, is a histone demethylase containing Jumonji C (*JmjC*) domain, and its function depends on ferrous ion (Fe [II]) and α-Ketoglutarate (α-KG) as prosthetic groups, and has demethylation activity on lysine modified by one-two-trimethylation through hydroxylation [11]. An increasing amount of evidence shows that the *JmjC* protein is an important histone lysine demethylase family, which plays an important role in maintaining stable histone methylation in the body [10,11].

The *JmjC* gene family has been comprehensively identified in several plant species, including *Zea mays* [12], *Glycine max* [13], and *Oryza sativa* [14]. Twenty-one *JMJ* gene family members have been identified in Arabidopsis; these proteins are involved in RNA silencing, DNA methylation, and the regulation of brassinosteroid (BR) signaling pathways. They have important functions such as influencing flowering, biological rhythms, and bud regeneration [15]. At present, according to sequence similarity and catalytic specificity, Arabidopsis *JmjC* genes can be divided into five categories: KDM4/JHDM3 (AtJMJ11-13), KDM5/JARID (AtJMJ14-19), JMJD6 (AtJMJ21/22), KDM3/JHDM2 (AtJMJ24-29), and the *JmjC* domain-only (AtJMJ20 and AtJMJ30-32) subfamily [11,15,16]. Members of different subfamily acting on different substrates can cause varying degrees of impact. Specifically, the KDM5/JARID subfamily members can remove the methylation of H3K4me1/2/3 [17], the KDM4/JHDM3 subfamily can remove the methylation of H3K9me2/3 and H3K36me2/3, while the KDM3/JHDM2 subfamily can demethylate H3K9me1 and H3K9me2 [18]. JMJD6 subfamily proteins have H3K27me2/3 demethylation activity [11,19], and *JmjC* domain-only subfamily proteins can remove the methylation of H3K27me3 [20].

*JmjC*-like proteins participate in the regulation of numerous gene expression and chromatin activities through histone demethylation and interaction with other chromatin modifications, and involve biological processes such as development, metabolism, and environmental response [21,22,23]. Early Flowering 6 (ELF6/AtJMJ11) and Relative of Early Flowering 6 (REF6/AtJMJ12) in Arabidopsis both belong to the KDM4/JHDM3 subfamily, and have histone H3K9me2/3 and H3K27me2/3 demethylase activities, but the two have completely opposite functions in controlling flowering time [24]. AtJMJ15 and AtJMJ18 belong to the KDM5/JARID subfamily, which can bind *FLOWERING LOCUS C* (*FLC*) chromatin. The overexpression of *AtJMJ15* and *AtJMJ18* reduces the level of H3K4 methylation in *FLC* chromatin and inhibits *FLC* expression, thereby promoting the expression of *FLOWERING LOCUS T (FT)* in accompanying cells, stimulating flowering, and leading to a significant early flowering phenotype [25,26]. The zinc finger protein SOMNUS (SOM) is an important seed germination inhibitor [27]. *JMJ22* is a member of the JMJD6 subfamily, and its expression with *JMJ20* can be directly inhibited by SOM to control seed germination [28]. Rice JMJ706 and Arabidopsis IBM1/JMJ25 belong to the KDM3/JHDM2 subfamily and have been shown to remove H3K9 methylation [29,30]. Although *JMJ24* belongs to the same subfamily as *IBM1*/*JMJ25*, *JMJ24* may have the function of counteracting *IBM1*/*JMJ25* in gene regulation [31]. *AtJMJ30* is a member of the *JmjC* domain-only subfamily. It is a component of the plant biological clock and participates in the regulation of the circadian rhythm, mainly by participating in the regulation of the length of the circadian rhythm [32]. In addition, the *JmjC* genes also play a vital role in plant response to stress. In Arabidopsis, compared with wild-type, plants overexpressing *AtJMJ15* shows stronger salt tolerance, while mutants with loss of function are more sensitive to salt stress [33]. In the rice *jmj704* deletion mutant, the level of H3K4me2/3 is significantly increased. *JMJ704* suppresses the negative regulation of rice plants’ defense by reducing the activation marker H3K4me2/3 on it [34]. Overexpression of *OsJMJ705* reduces the methylation level of H3K27me2/3, which results in preferential activation of H3K27me3-labeled biotic stress response genes, and enhances rice plants’ resistance to bacterial leaf blight (*Xanthomonas oryzae* pv. *oryzae*) [35]. In rice, JMJ706 can specifically remove the demethylation of histone H3K9me2 and participate in the response to salt, drought, ABA, and paraquat stress; the response to paraquat stress is more obvious. The loss-of-function mutant plants of *JMJ706* have enhanced resistance to paraquat and hydrogen peroxide [36].

However, the evolution of the histone demethylase gene containing the *JmjC* domain in plants is still unclear and requires further investigation. In this study, *JmjC* genes in various green plants were identified, and an evolutionary analysis of the whole genome was performed. We focused on the identification of *JmjC* genes in different species, the evolutionary relationship, and an analysis of conserved domains and promoter elements. The results of this study provide a theoretical basis for the evolution and biological functions of the *JmjC* genes in plants.

## 2. Results

### 2.1. Identification of JmjC Genes in Green Plants

**Figure 1:**
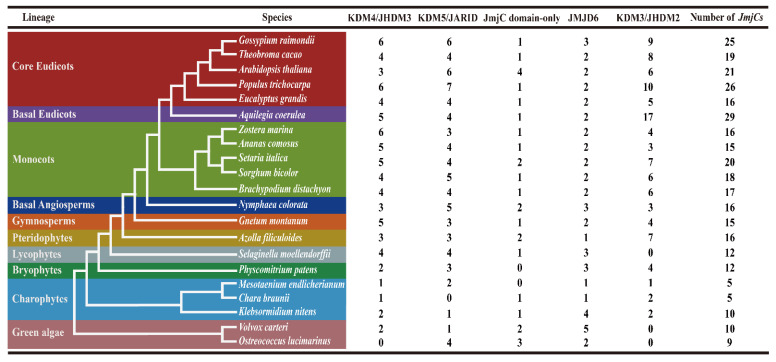
Phylogenetic relationships among 21 species. Different background colors in the phylogenetic tree show different pedigrees. The right side shows the number of *JmjC* genes in each species of the different subfamilies and the total number of *JmjC* genes found in the genome of each species.

In order to comprehensively understand the *JmjC* genes in green plants, 21 plant species, which have published genome-wide data, were used to screen the *JmjC* genes. A total of 332 *JmjC* family genes were identified, and each sequence contained a conserved domain of *JmjC* (Table S1). These 21 species represent 10 plant evolutionary lineages, including green algae (*Ostreococcus lucimarinus* and *Volvox carteri*), charophytes (*Mesotaenium endlicherianum*, *Chara braunii*, and *Klebsormidium nitens*), bryophytes (*Physcomitrium patens*), lycophytes (*Selaginella moellendorffii*), pteridophytes (*Azolla filiculoides*), gymnosperms (*Gnetum montanum*), basal angiosperms (*Nymphaea colorata*), monocots (*Brachypodium distachyon*, *Sorghum bicolor*, *Setaria italica*, *Ananas comosus*, and *Zostera marina*), basal eudicots (*Aquilegia coerulea*), and core eudicots (*Gossypium raimondii*, *Theobroma cacao*, *Arabidopsis thaliana*, *Populus trichocarpa*, and *Eucalyptus grandis*) (Figure 1). The *JmjC* gene family has already appeared in green algae, with 9 and 10 *JmjC* genes in *O*. *lucimarinus* and *V*. *carteri*, respectively. The copy number of *JmjC* genes varied widely in other plants, ranging from 5 in *C. braunii* and *M*. *endlicherianum* to 29 in *A*. *coerulea*. Specifically, 10 *JmjC* genes were found in *K*. *nitens*, 12 in *P*. *patens* and *S*. *moellendorffii*, 15 in *G*. *montanum* and *A*. *comosus*, while there were 16 in *A*. *filiculoides*, *N*. *colorata*, *Z*. *marina*, and *E*. *grandis*, 17 in *B*. *distachyon*, 18 in *S*. *bicolor*, 19 in *T*. *cacao*, 20 in *S*. *italica*, 21 in *A*. *thaliana*, 25 in *G*. *raimondii* [37], and 26 in *P*. *trichocarpa*, which indicated that *JmjC* family members had expanded largely in the process of evolution.

### 2.2. Phylogenetic Evolution Analysis of Plant JmjC Genes

**Figure 2:**
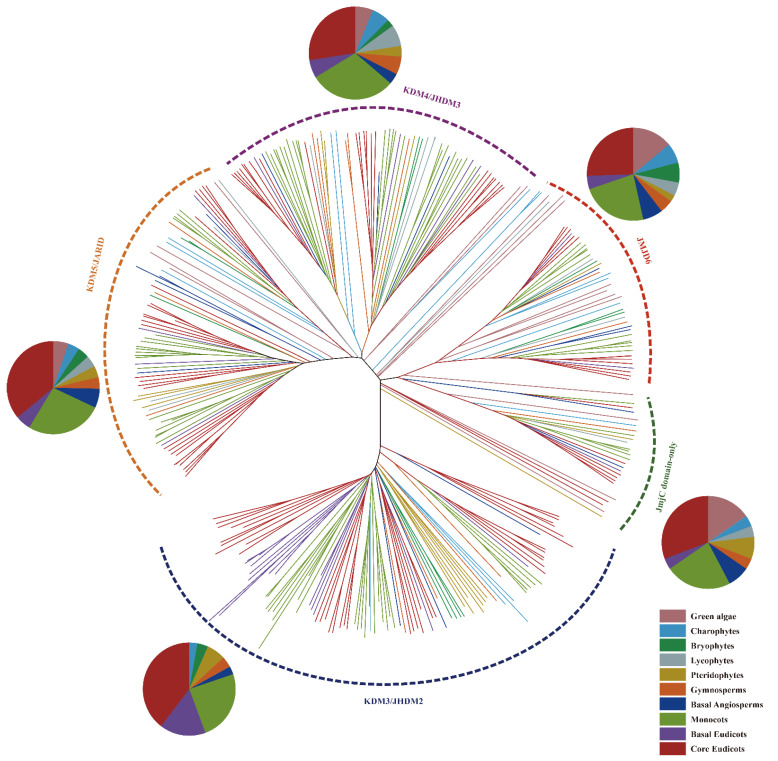
Phylogenetic tree of *JmjC* proteins in 21 plant species. *JmjC*s are divided into five subfamilies, which are represented by arcs of different colors. The lines of different colors in the evolutionary tree represent the *JmjC*s of 10 different lineages. The color of the pie chart in the figure corresponds to the color of the *JmjC*s in the evolutionary tree.

In order to further study the evolutionary relationship between *JmjC* proteins in green plants, a multiple sequence alignment was performed on the full-length amino acid sequences of 332 *JmjC*s, and the neighbor-joining (NJ) phylogenetic tree was constructed using MEGA 7.0 software. As shown in Figure 2, all *JmjC* genes formed five branches, namely, KDM4/JHDM3, KDM5/JARID, *JmjC* domain-only, JMJD6, and KDM3/JHDM2 (Table S2). From the pie chart analysis, it can be clearly seen that most of the *JmjC* genes in all subfamilies were present in eudicotyledonous and monocotyledonous plants (Figure S1). Moreover, there were differences in the number of *JmjC* genes among different subfamilies. For example, the KDM3/JHDM2 subfamily had the largest number of *JmjC* family members, a total of 104, but no members of green algae of lycophytes belonged to the subfamily. Additionally, the number of KDM3/JHDM2 subfamily members varied the most, ranged from 0 to 17 (*A*. *coerulea*). Additionally, the number of *JmjC* genes in the *JmjC* domain-only subfamily was the least, only 28 in total, and the majority contained four genes in *A*. *thaliana*. In chara (*M*. *endlicherianum*) and bryophytes (*P. patens*), no members of the *JmjC* domain-only subfamily were found, and there were three genes in green algae (*O*. *lucimarinus*); two genes in *V*. *carteri*, *A*. *filiculoides*, *N. colorata*, and *S*. *italica*; and only one *JmjC* gene in the other plants. Except for the KDM3/JHDM2 and *JmjC* domain-only branches, the remaining branches contained the *JmjC* genes of all the lineages analyzed. There were a total of 75 *JmjC* genes in the KDM4/JHDM3 branch, and there were similar numbers of genes in eudicotyledons and monocots, 23 and 24 *JmjC* genes, respectively, but no genes from *O*. *lucimarinus* belonged to this family. There were a total of 77 *JmjC* genes in the KDM5/JARID branch; among them, no genes were found in *C. braunii*, and 27 and 20 genes were found in eudicotyledons and monocots, respectively. There were 48 *JmjC* genes in the JMJD6 branch (Table S3).

### 2.3. Analysis of Conserved Amino Acid Residues of JmjC

**Figure 3:**
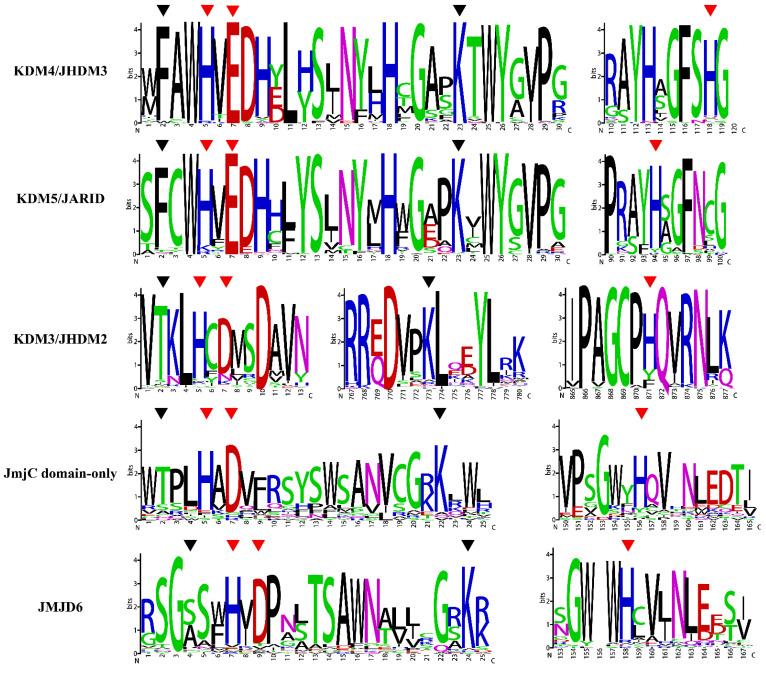
Conserved amino acid residues of *JmjC* protein in plants. The height of each letter in the figure indicates the degree of conservation of amino acid residues. The number on the abscissa indicates the relative position of the amino acid in the motif. The three conserved catalytic residues of Fe (II) (His 188, Glu/Asp 190, and His 276) are marked with red triangles. The two conserved catalytic residues of α-KG (Ther/Phe 185 and Lys 206) are marked with black triangles.

Jumonji C (*JmjC*) domain demethylates histones through an oxidative mechanism that requires ferrous ion (Fe [II]) and α-ketoglutarate (α-KG) as cofactors in addition to the hydroxylation of proteins [38]. The three residues that bind to Fe (II) (His 188, Glu/Asp 190, and His 276), and two residues that bind to α-KG (Thr/Phe 185 and Lys 206) are highly conserved and play an important role in the function of enzyme activity. To further assess the conservation of the *JmjC* domain, a multiple sequence alignment of *JmjC* domains in 21 plants species of different subfamilies was performed, and the results were displayed as sequence logos. As shown in Figure 3, the conserved residues varied within the subfamily. The subfamilies KDM4/JHDM3 and KDM5/JARID contained the original sites, namely, His (H), Glu (E), and His (H) and Phe (F) and Lys (K), respectively. The residues of subfamilies KDM3/JHDM2 and *JmjC* domain-only changed to His (H), Asp (D) and His (H) and Thr (T) and Lys (K), respectively. In addition, the first residue of the JMJD6 subfamily was replaced by Ser (S)/Ala (A), and the remaining four sites were conserved. Among the five subfamilies, the *JmjC* domain-only subfamily had the largest variation in amino acids, followed by the JMJD6 subfamily. Compared with the other four subfamilies, the amino acid residues of the KDM3/JHDM2 subfamily were farther apart. The conservation of the interaction site was related to the demethylase activity of the *JmjC* gene, suggesting that functional differentiation of the *JmjC* gene may have occurred in the process of evolution.

### 2.4. Analysis of Cis-Acting Elements in the Promoter Regions of JmjC Genes

**Figure 4:**
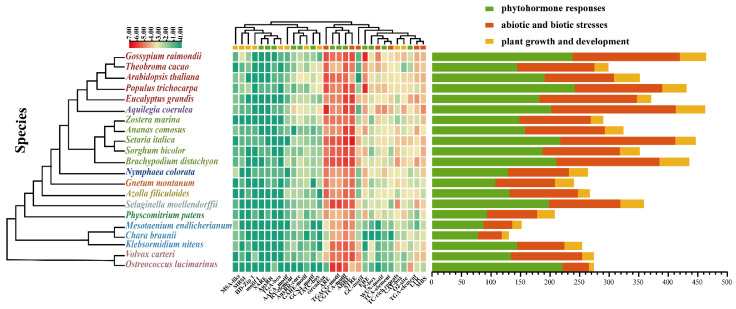
Analysis of promoter *cis*-elements of *JmjC* genes in plants. The left side of the heatmap is the evolutionary history of 21 species. Green, red, and yellow boxes represent *cis*-elements related to phytohormone responses, biotic and abiotic stresses, and plant growth and development, respectively. The histogram indicates the number of *cis*-acting elements of different types in each species.

Recent research has shown that *JmjC* genes have biological functions in plant growth and development, hormone response, and abiotic stress [39]. Therefore, we analyzed the *cis*-acting elements of the 2000 bp upstream regions from the transcription start site (TSS) of all the selected *JmjC* genes to explore their potential function. Finally, three types of *cis*-acting elements related to phytohormone response, abiotic and biotic stress response, and plant growth and development were detected (Figure 4 and Table S4). The results revealed six types of phytohormone-responsive regulatory elements that were identified in the *JmjC* promoter regions, including abscisic acid (ABA)-responsive elements (ABRE), gibberellin (GA)-responsive elements (GARE-motif, TATC-box, and P-box), methyl jasmonate (MeJA)-responsive elements (TGACG-motif and CGTCA-motif), salicylic acid (SA)-responsive elements (TCA-element and SARE), auxin (IAA)-responsive elements (TGA-element, AuxRR-core, TGA-box, and AuxRR), and ethylene-responsive elements (ERE). Among them, the elements associated with ABA and MeJA were abundant in all the species, which are major signaling molecules affecting plant growth and stress responses [40]. Furthermore, seven types of stress-response elements, namely, LTR, MBS, ARE, GC-motif, WUN-motif, TC-rich repeats, and STRE, which are responsible for low-temperature stress response, drought stress response, anaerobic induction, enhancing anoxic specific inducibility, wound stress response, defense stress response, and stress response, respectively, were identified. The most common elements that related to abiotic and biotic stress response were ARE and STRE. The *cis*-acting element related to plant growth and development mainly included the cellular development and metabolism regulation categories. To be specific, CAT-box, GCN4_motif, MSA-like, RY-element, AACA_motif, motif I, and HD-Zip 1 are associated with cell differentiation and tissue development. While circadian, O_2_-site, and MBSI are associated with circadian control, zein metabolism regulation, and flavonoid biosynthesis gene regulation, respectively. These analyses showed that there were numerous different *cis*-acting elements in the promoter of *JmjC* genes, suggesting that they might not only participate in the transduction of hormone signals, but also in the response to external pressure.

### 2.5. Expression Profiles of JmjC Genes in Different Tissues

**Figure 5:**
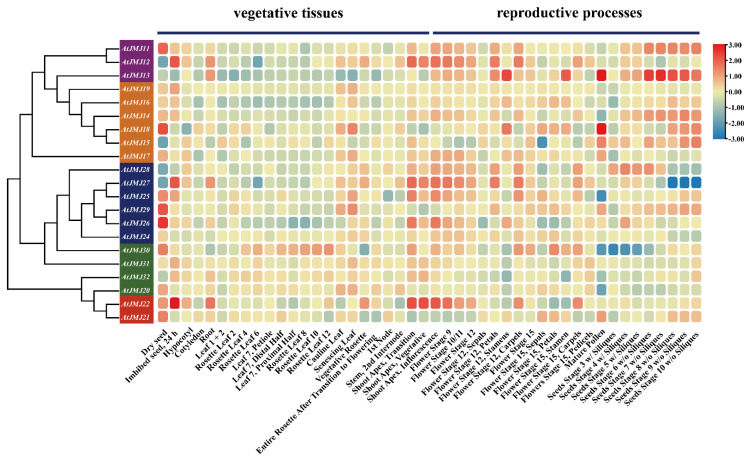
Expression profiles of the *JmjC* genes in different tissues and developmental stages of *A. thaliana*. On the left is the phylogenetic tree of Arabidopsis *JmjC*s.

To investigate the role of plant *JmjC* genes in different stages of tissue development, RNA-seq data were used to study the expression profile of *JmjC* genes in model plant *A. thaliana*. The transcriptome data of *A. thaliana* revealed that *JmjC* genes had different expression patterns in different tissues (Figure 5), suggesting that these genes had different functions in various developmental stages. In 23 vegetative tissues, most *AtJMJ*s had high expression in dry seed, cauline leaf, senescing leaf, and shoot apex. *AtJMJ12*, *AtJMJ22*, and *AtJMJ27* were highly expressed in seed imbibed for 24 h. Moreover, most *AtJMJ*s had higher expression levels in tissues during the reproductive processes than that in vegetative tissues. In particular, *AtJMJ13* was highly expressed only during the reproductive stage. The expression levels of *AtJMJ*s in the shoot apex; inflorescence; and in flowering stages 9, 10/11, and 12 were relatively high. Some genes were expressed significantly only at certain stages, for example, *AtJMJ14* was highly expressed only in the seed stages, and *AtJMJ15* only in the mature pollen and seed stages. Additionally, there were individual genes, such as *AtJMJ16*, that had low expression levels in all the tissues selected. The same subfamily may have different gene expression patterns: *AtJMJ22* was highly expressed in various tissues, while *AtJMJ21* only in dry seed.

### 2.6. Interaction of the JmjC Protein in Plants

**Figure 6:**
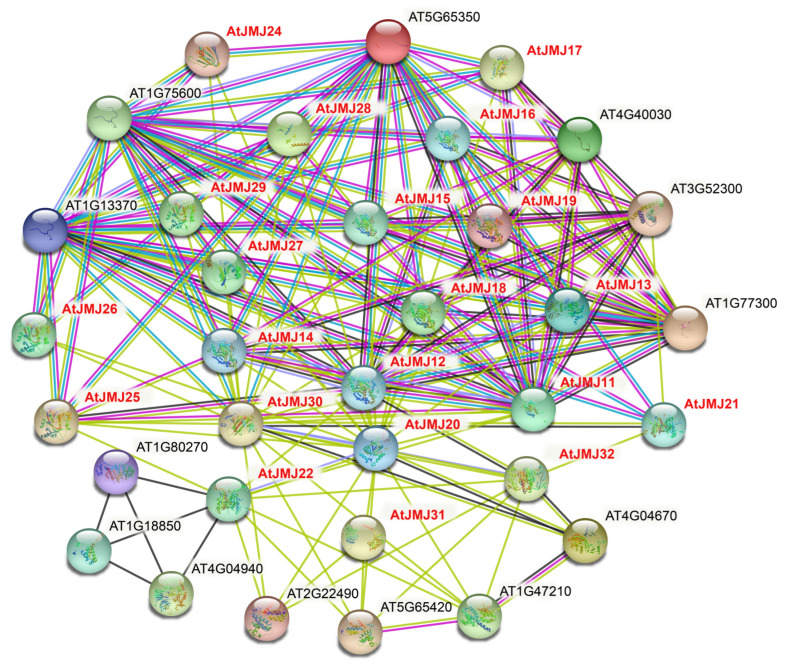
Functional network assembly of the *A. thaliana JmjC* proteins. Red letters are the *A. thaliana* protein. Light blue and purple lines represent known interactions determined by the database and experiments, respectively. Green, red, and blue lines represent predicted interactions from gene proximity, fusion, and symbiosis, respectively. Light green, black, and gray lines indicate other interactions from text mining, co-expression, and protein homology, respectively. Empty nodes: proteins of unknown three-dimensional structure. Filled nodes: some three-dimensional structures are known or predicted.

To further detect the potential roles of *JmjC* family members, we investigated the interaction of the *JmjC* protein in *A. thaliana* using the STRING database (Figure 6). The results showed that the interaction network of the *JmjC* protein exhibited complex functional relationships. For instance, the majority of *A. thaliana JmjC* proteins interacted with the histone superfamily proteins (AT5G65350.1/HTR11, AT5G10980.1/HTR4, AT1G75600.1/AT1G75600, AT1G13370.1/AT1G13370), and AT5G19840/AtJMJ31 interacted with cyclins (AT5G65420.3/CYCD4;1, AT2G22490.2/CYCD2;1. AT1G47210.2/CYCA3;2). Moreover, AtJMJ11/AT5G04240, AtJMJ12/AT3G48430 and AtJMJ13/AT5G46910 interacted with each other, and they all interacted with methyltransferase-associated proteins (AT1G77300.1/EFS), suggesting that the *JmjC* protein is involved in many biological processes through protein interactions.

## 3. Discussion

The plant *JmjC* gene family is an important class of histone lysine demethylation enzymes, which have important effects on plant growth and development. In this study, a total of 332 *JmjC* genes were identified in 21 green plants, and each sequence contained a *JmjC* conserved domain. According to the analysis of species evolution (Figure 1), the *JmjC* genes existed in all the analyzed plants, and the copy number of the *JmjC* genes varied widely, ranging from 5 in *C. braunii* and *M. endlicherianum* to 29 in *A. coerulea*, indicating that the *JmjC* family genes presented a wide range of changes in the course of plant evolution. The phylogenetic relationship of plant *JmjC*s indicated (Figure 2) that among the five subfamilies, the KDM3/JHDM2 subfamily appeared in charophytes first, and no *JmjC* genes of this subfamily were found in *S. moellendorffii*. Additionally, no *JmjC* domain-only subfamily members were found in either *M. endlicherianum* or *P. patens*, no KDM4/JHDM3 members were found in *O*. *lucimarinus*, and no KDM5/JARID members were found in *C. braunii*, which indicated that the *JmjC* genes of this subfamily in these plants were lost during evolution. Only the JMJD6 subfamily of the *JMJC* genes were retained in all the plant species. The five amino acid residues within the cofactor binding sites predicted by the *JmjC* domain were conserved and are important for the enzyme activity of the *JmjC* protein [11,15]. Among them, three residues bind to the Fe (II) cofactor, while α-ketoglutarate (α-KG) requires another two residues [15]. The five amino acid residues were highly conserved in the KDM4/JHDM3, KDM5/JARID, KDM3/JHDM2, and *JmjC* domain-only subfamilies. However, the JMJD6 subfamily lost a key α-KG binding residue (F/T) and was replaced by S/A (Figure 3). Mutations in amino acids and changes in the distance between residues may lead to changes in plants’ *JmjC* protein function.

The promoter is a DNA sequence that is located in the upstream region of the 5′ end of the structural gene, which can activate RNA polymerase, making it accurately bind to the template DNA and have specificity for transcription initiation [41]. The specificity of gene expression depends on *cis*-regulatory elements and their association with the *JmjC* genes [42]. Therefore, 2000 bp sequences upstream of the 5′ end of all the *JmjC* genes were extracted, and the *cis*-acting elements in the promoter regions were predicted using the PlantCRAE online tool (Figure 4). The results showed that many *cis*-acting elements in response to plant hormones, biotic and abiotic stresses, and plant growth and development were included. ABRE is an ABA-responsive *cis*-acting element. Under the condition of water shortage, the ABA content in plant leaves increases and causes stomatal closure, so it can be used as an anti-transpiration agent. In addition, drought, cold, high temperatures, salinization, and waterlogging stress can rapidly increase ABA in plants and enhance stress resistance. For example, ABA can induce the re-synthesis of certain enzymes and increase the cold, flood, and salt resistance of plants [41,42,43]. Methyl jasmonate is involved in the germination, rooting, flowering, and senescence of plants, which is also thought to be a defense mechanism that positively responds to most biotic and abiotic stresses in plants [44]. Additionally, there were amounts of *cis*-elements related to anaerobic induction (ARE) and stress response (STRE). The *cis*-acting elements associated with the cellular development and metabolism regulation categories were common in the promoter regions in all the species. Therefore, it was speculated that the *JmjC* family genes play an important role in plant growth and stress response.

The potential role of the *JmjC* family members was further explored by predicting Arabidopsis transcriptome data and protein interaction networks (Figure 5 and Figure 6). Most *AtJMJ*s had higher expression levels in tissues during the reproductive processes than that in vegetative tissues, suggesting that they may play a more important role in the reproductive stage. Meanwhile, the protein interaction prediction indicated that AtJMJ13/At5G46910 interacted with the AtJMJ11/At5G04240 and AtJMJ12/At3G48430 proteins, and both of them interacted with methyltransferase-related proteins. Additionally, the mutant of *AtJMJ11/At5G04240* leads to an early flowering phenotype in Arabidopsis [45]. The overexpression of *ATJMJ12/AT3G48430* shows a pleiotropic phenotype of early flowering, short stature, and various degrees. Furthermore, the analysis of the cellular and molecular levels has found that the overexpression of *AtJMJ12/At3G48430* results in a decrease in the cells of the leaf surface [46]. In addition, studies have shown that AtJMJ13/AT5G46910 is an Arabidopsis H3K27Me3 demethylase and a temperature photoperiod-dependent inhibitory factor for flowering. The Arabidopsis *jmj13* mutant blooms early under short-day and high-temperature conditions [47]. Therefore, Arabidopsis *JmjC* proteins play a major role in plant growth and development.

## 4. Materials and Methods

### 4.1. Genome-Wide Identification of JmjC Family Genes in Plants

In this study, the genome-wide data of 21 plant species were analyzed, and 21 *A. thaliana JmjC* genes were downloaded from TAIR (https://www.arabidopsis.org/ (accesses on 18 August 2021)) [48]. The sources of genomic data for other species are listed in Table S5. To identify *JmjC* family genes in the selected species, BlastP and tBlastN searches [49] were applied to search the genome data with *JmjC* protein sequences in *A. thaliana* as queries. All the identified candidates were submitted to the Pfam [50] and SMART [51] databases, and genes encoding proteins containing a conserved protein domain (PF02373) were identified as *JmjC* genes.

### 4.2. Evolutionary Analysis 

The ClustalW [52] program was used for the multiple sequence alignment of all the *JmjC* protein sequences in 21 plant species with the default parameters. Then, a phylogenetic tree was constructed using the MEGA 7.0 [53] with the neighbor-joining (NJ) method. The reliability was assessed with the *p*-distance model and 1000 bootstrap replications. Excel was used to count the number of genes contained in each *JmjC* subfamily in different species, and Origin 8.0 software [54] was used to make a pie chart.

### 4.3. Analysis of Conserved Amino Acid Residues of JmjC

Multiple sequence alignments were performed for each subfamily of *JmjC* protein sequences using the ClustalX 2.1 [55] software with the default parameters to obtain the conserved regions. Sequence logos for the conserved domains in *JmjC*s were generated using the WebLogo (http://weblogo.berkeley.edu/logo.cgi (accesses on 4 February 2022)) online tool [56].

### 4.4. Analysis of Cis-Acting Elements in the Promoter Regions of JmjC Genes

The 2000 bp upstream regions from the transcription start sites (TSS) of all the *JmjC* genes were obtained from the Phytozome database [57] and genome files, and then the PlantCARE (http://bioinformatics.psb.ugent.be/webtools/plantcare/html (accesses on 4 February 2022)) online tool [58] was used to search for *cis*-acting elements. The results were classified and counted [59], and finally visualized using the TBtools software [60].

### 4.5. Expression Pattern of Plant JmjC Genes in Different Tissues

Open expression data of various tissues in *A. thaliana* were acquired from the Bio-Analytic Resource for Plant Biology public database (http://bar.utoronto.ca/ (accesses on 4 February 2022)) [61], based on *JmjC* IDs of *A. thaliana*, including 23 vegetative tissues and 23 tissues during reproductive processes. A heatmap generated by the TBtools software [60] was used to display the relative expression levels.

### 4.6. Prediction of JmjC Protein Interactions

The prediction of Arabidopsis *JmjC* protein interactions was built using the STRING database (https://cn.string-db.org/ (accessed on 10 February 2022)) [62], with the confidence parameter set at a threshold of 0.40 and the number of interactions set to 5; the other parameters were as default.

## 5. Conclusions

In this study, a total of 332 *JmjC* genes were identified from 21 different plants. The evolutionary analysis suggested that *JmjC* genes had already appeared in algae, and the conserved domain analysis showed that each sequence contained a typical *JmjC* conserved domain, indicating that it was strongly conserved in plant evolution. The phylogenetic analysis demonstrated that the genes of the KDM3/JHDM2 subfamily might have emerged during the transition from water to land. Additionally, the analysis of the *cis*-acting elements showed that the *JmjC* genes were rich in *cis*-acting elements in response to plant hormones and biotic and abiotic stresses. The transcriptome data and protein interaction analysis indicated that *JmjC* genes play an important role in plant growth and development.

## Data Availability

The data presented in this study are available in the Supplementary Materials.

## Supplementary Materials

The following supporting information can be downloaded at: https://www.mdpi.com/article/10.3390/plants11081041/s1, Refs [63,64,65,66,67,68,69,70,71,72,73,74,75,76,77,78,79,80,81], Table S1: JmjC gene IDs in 21 species; Table S2: Species selection and subfamily distribution in the plant JmjC gene phylogenetic tree; Table S3: JmjC gene family distribution; Table S4: List of identified cis-elements in the putative promoter region of JmjC genes; Table S5: The genome data sources for the species used in this study; Figure S1: JmjC gene family classification.
