# Supplementary figures and images for: Evolutionary History and Functional Diversification of the JmjC Domain-Containing Histone Demethylase Gene Family in Plants
Source: Plants (Basel). 2022 Apr 12;11(8):1041. doi: 10.3390/plants11081041 (PMC9029850; doi:10.3390/plants11081041)

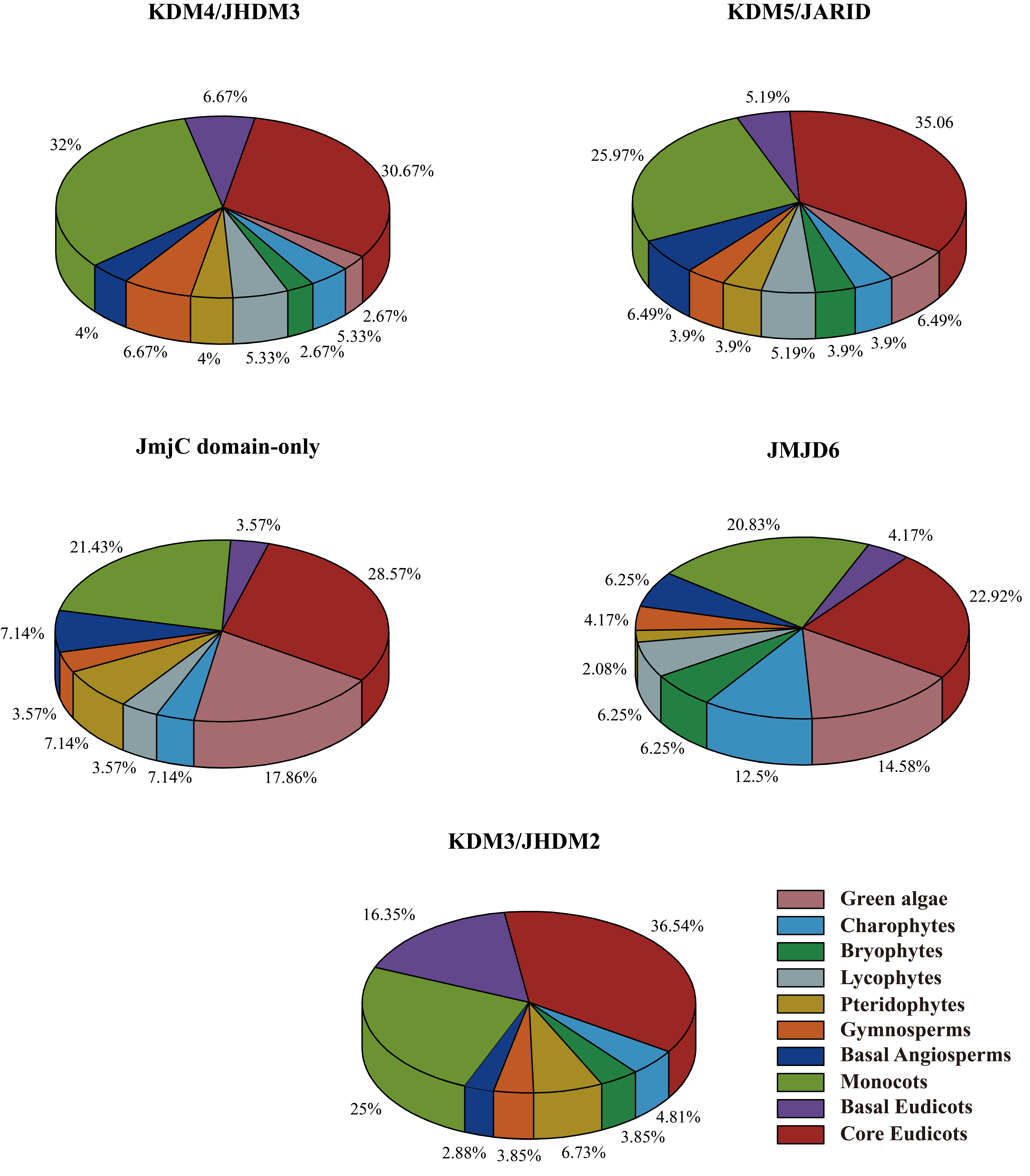

Supplement: Supplementary file 1 [file plants-11-01041-s001.zip › Figure S1. JmjC gene family classification.tif]
